# Supplementary material for: Associations of heavy metals and urinary sodium excretion with obesity in adults: A cross-sectional study from Korean Health Examination and Nutritional Survey
Source: PLoS One. 2025 Jan 31;20(1):e0317190. doi: 10.1371/journal.pone.0317190 (PMC11785309; doi:10.1371/journal.pone.0317190)
Supplement: S1 Table — (DOCX) [file pone.0317190.s001.docx]

**Supplementary Table 1.** OR (95% CI)^a^ of overweight and obesity by the levels of heavy metals and 24-hour sodium excretion ^b^ in the healthy general population (N = 6,137)^c^

|  | **Normal**  **(N=3,979)** | **Underweight**  **(N=287)** | **Underweight** | **Overweight & Obesity**  **(N=1,878)** | **Overweight & Obesity** |
| --- | --- | --- | --- | --- | --- |
|  | N (%) | N (%) | OR (95% CI) | N (%) | OR (95% CI) |
| **Serum cadmium levels (µg/L)** | | | | | |
| Low (< 1.0) | 1996 (50.2) | 185 (64.5) | Ref | 884 (47.1) | Ref |
| High (≥ 1.0) | 1976 (49.7) | 102 (35.5) | 0.82 (0.57-1.17) | 994 (52.9) | 1.03 (0.87-1.21) |
| 1T (< 0.7) | 1324 (33.3) | 140 (48.8) | Ref | 578 (30.8) | Ref |
| 2T (0.7-1.3) | 1343 (33.8) | 78 (27.2) | 0.71 (0.49-1.02) | 630 (33.5) | 1.01 (0.84-1.21) |
| 3T (≥ 1.4) | 1305 (32.8) | 69 (24.0) | 0.81 (0.51-1.29) | 670 (35.7) | 1.01 (0.83-1.22) |
| **Serum mercury levels (µg/L)** | | | | | |
| Low (< 3.9) | 2098 (52.8) | 187 (65.2) | Ref | 783 (41.7) | Ref |
| High (≥ 3.9) | 1874 (47.2) | 100 (34.8) | 1.09 (0.78-1.53) | 1095 (58.3) | **1.48 (1.27-1.72)** |
| 1T (< 3.0) | 1394 (35.1) | 130 (45.3) | Ref | 519 (27.6) | Ref |
| 2T (3.0-5.0) | 1358 (34.2) | 101 (35.2) | 1.12 (0.81-1.56) | 591 (31.5) | 1.04 (0.86-1.26) |
| 3T (≥ 5.1) | 1220 (30.7) | 56 (19.5) | 0.91 (0.59-1.40) | 768 (40.9) | **1.50 (1.24-1.82)** |
| **Urinary arsenic excretion levels (mcg/L)** | | | | | |
| Low (< 111.4) | 900 (49.9) | 78 (54.2) | Ref | 401 (49.5) | Ref |
| High (≥ 111.4) | 904 (50.1) | 66 (45.8) | 1.21 (0.75-1.95) | 409 (50.5) | 0.93 (0.76-1.14) |
| 1T (< 80.1) | 595 (33.0) | 51 (35.4) | Ref | 272 (33.6) | Ref |
| 2T (80.1 – 154.3) | 594 (32.9) | 55 (38.2) | 1.39 (0.83-2.33) | 272 (33.6) | 0.90 (0.69-2.21) |
| 3T (≥ 154.3) | 615 (34.1) | 38 (26.4) | 1.19 (0.64-2.21) | 266 (32.8) | 0.87 (0.67-1.13) |
| **Urinary arsenic-creatinine ratio (µg /mg)** | | | | | |
| Low (< 0.8) | 907 (50.3) | 80 (55.6) | Ref | 409 (50.5) | Ref |
| High (≥ 0.8) | 897 (49.7) | 64 (44.4) | 1.06 (0.66-1.69) | 401 (49.5) | 0.92 (0.73-1.16) |
| 1T (< 0.5) | 598 (33.1) | 65 (45.1) | Ref | 282 (34.8) | Ref |
| 2T (0.5 – 1.3) | 605 (33.5) | 36 (25.0) | 0.84 (0.50-1.43) | 270 (33.3) | 0.91 (0.70-1.18) |
| 3T (≥ 1.3) | 601 (33.3) | 43 (29.9) | 1.04 (0.58-1.85) | 258 (31.8) | 0.82 (0.63-1.08) |
| **Urinary 24-hour sodium excretion levels (mg/day)** | | | | | |
| Low (< 3233.6) | 2106 (43.0) | 215 (74.9) | Ref | 747 (39.8) | Ref |
| High (≥ 3233.6) | 1866 (47.0) | 72 (25.1) | **0.56 (0.39-0.81)** | 1131 (60.2) | **1.72 (1.46-2.02)** |
| 1T (< 2885.6) | 1402 (35.3) | 180 (62.7) | Ref | 461 (24.5) | Ref |
| 2T (2885.6 – 3588.7) | 1375 (34.6) | 68 (23.7) | **0.47 (0.32-0.68)** | 607 (32.3) | **1.29 (1.06-1.57)** |
| 3T (≥ 3588.8) | 1195 (30.1) | 39 (13.6) | **0.41 (0.26-0.66)** | 810 (43.1) | **2.12 (1.75-2.57)** |

Abbreviation: T, Tertile; OR, Odds ratio.

a. Adjusted for age, sex, household income, education level, marital status, smoking status, physical activity, dietary potassium, dietary energy intake, history of diabetes and hypertension.

b. Urinary 24-hour sodium excretion levels were estimated by Tanaka equation in a spot urine.

c. The Korean Health Examination and Nutritional Survey [KHEANS], 2008-2012
