# Supplementary material for: Associations of heavy metals and urinary sodium excretion with obesity in adults: A cross-sectional study from Korean Health Examination and Nutritional Survey
Source: PLoS One. 2025 Jan 31;20(1):e0317190. doi: 10.1371/journal.pone.0317190 (PMC11785309; doi:10.1371/journal.pone.0317190)
Supplement: S2 Table — (DOCX) [file pone.0317190.s002.docx]

**Supplementary Table 2.** OR (95% CI) ^a^ of overweight and obesity by the combination of serum mercury and urinary 24-hour sodium excretion ^b^ in the general population (N = 6,137)^c^

| **Urinary sodium levels** | **Serum mercury levels** | **Normal**  **(N=4,225)** | **Underweight**  **(N=291)** | **Underweight** | **Overweight & Obesity**  **(N=2,093)** | **Overweight & Obesity** |
| --- | --- | --- | --- | --- | --- | --- |
|  |  | N (%) | N (%) | OR (95%CI) | N (%) | OR(95%CI) |
| 1T | 1T | 544 (13.7) | 88 (30.7) | Ref | 132 (7.0) | Ref |
| 2T | 1T | 465 (11.7) | 58 (20.2) | 0.97 (0.61-1.54) | 142 (7.6) | 1.10 (0.76-1.60) |
| 1T | 2T | 393 (9.9) | 34 (11.8) | 0.99 (0.56-1.75) | 187 (10.0) | **1.67 (1.15-2.43)** |
| 2T | 2T | 472 (11.9) | 30 (10.4) | **0.45 (0.27-0.76)** | 186 (9.9) | **1.54 (1.06-2.23)** |
| 3T | 1T | 474 (11.9) | 25 (8.7) | 0.54 (0.29-1.00) | 179 (9.5) | 1.21 (0.83-1.76) |
| 1T | 3T | 429 (10.8) | 13 (4.5) | **0.39 (0.18-0.83)** | 242 (12.9) | **2.11 (1.46-3.04)** |
| 3T | 2T | 378 (9.5) | 12 (4.2) | **0.28 (0.12-0.62)** | 201 (10.7) | **2.13 (1.47-3.09)** |
| 2T | 3T | 419 (10.5) | 18 (6.3) | 0.62 (0.32-1.21) | 270 (14.4) | **2.60 (1.85-3.65)** |
| 3T | 3T | 398 (10.0) | 9 (3.1) | **0.32 (0.12-0.89)** | 339 (18.0) | **3.12 (2.22-4.39)^d^** |
|  |  |  |  |  |  |  |
| Low | Low | 1172 (29.5) | 146 (50.9) | Ref | 313 (16.7) | Ref |
|  | High | 934 (23.5) | 69 (24.0) | 1.06 (0.70-1.61) | 434 (23.1) | **1.62 (1.29-2.04)** |
| High | Low | 926 (23.3) | 41 (14.3) | **0.53 (0.33-0.84)** | 470 (25.0) | **1.87 (1.47-2.37)** |
|  | High | 940 (23.7) | 31 (10.8) | **0.65 (0.39-1.10)** | 661 (35.2) | **2.57 (2.05-3.22)** |

Abbreviation: T, Tertile ; OR, Odds ratio.

a. Adjusted for age, sex, household income, education level, marital status, smoking status, physical activity, dietary potassium, dietary energy intake, history of diabetes and hypertension.

b. Urinary 24-hour sodium excretion levels were estimated by Tanaka equation in a spot urine.

c. The Korean Health Examination and Nutritional Survey [KHEANS], 2008-2012

d. P-interaction: p < 0.01
