# Supplementary material for: Associations of heavy metals and urinary sodium excretion with obesity in adults: A cross-sectional study from Korean Health Examination and Nutritional Survey
Source: PLoS One. 2025 Jan 31;20(1):e0317190. doi: 10.1371/journal.pone.0317190 (PMC11785309; doi:10.1371/journal.pone.0317190)
Supplement: S3 Table — (DOCX) [file pone.0317190.s003.docx]

**Supplementary table 3.** Correlation matrix of independent variables in the study of heavy metals, urinary sodium excretion, and BMI levels.

|  | Age | Sex | Dietary Potassium intake | Dietary Water intake | Dietary Energy intake | Hypertension | Diabetes | Exercise | Urinary sodium excretion | Serum mercury | BMI |
| --- | --- | --- | --- | --- | --- | --- | --- | --- | --- | --- | --- |
| Age | 1.00 | 0.040*** | -0.134*** | -0.210*** | -0.169*** | -0.009 | 0.044*** | 0.035*** | 0.174*** | 0.092*** | 0.099*** |
| Sex | 0.040*** | 1.00 | -0.238*** | -0.214*** | -0.238*** | -0.014* | -0.001 | -0.002 | -0.021** | -0.239*** | -0.079*** |
| Dietary Potassium intake | -0.134*** | -0.238*** | 1.00 | 0.635*** | 0.530*** | -0.001 | -0.004 | 0.007 | 0.028*** | 0.115*** | 0.043*** |
| Dietary Water intake | -0.210*** | -0.214*** | 0.635*** | 1.00 | 0.527*** | 0.005 | -0.006 | 0.001 | 0.001 | 0.114*** | 0.015* |
| Dietary Energy intake | -0.169*** | -0.238*** | 0.530*** | 0.527*** | 1.00 | 0.011 | 0.026*** | 0.029*** | -0.003 | 0.078*** | 0.025*** |
| Hypertension | -0.009 | -0.014* | -0.001 | 0.011 | 0.005 | 1.00 | 0.086*** | 0.145*** | 0.003 | 0.004 | 0.001 |
| Diabetes | 0.044*** | -0.001 | -0.004 | 0.026*** | -0.006 | 0.026*** | 1.00 | 0.341*** | 0.036*** | 0.004 | -0.017* |
| Exercise | 0.035*** | -0.002 | 0.007 | 0.029*** | 0.001 | 0.029*** | 0.145*** | 1.00 | 0.044*** | -0.001 | -0.018*** |
| Urinary sodium excretion | 0.174*** | -0.021** | 0.028*** | -0.003 | 0.001 | -0.003 | 0.003 | 0.008 | 1.00 | 0.448*** | 0.218*** |
| Serum mercury | 0.092*** | -0.239*** | 0.115*** | 0.078*** | 0.114*** | 0.078*** | 0.004 | -0.001 | 0.044*** | 1.00 | 0.136*** |
| BMI | 0.099*** | -0.079*** | 0.043*** | 0.015* | 0.025*** | 0.001 | -0.017* | -0.018*** | 0.218*** | 0.136*** | 1.00 |

*p<0.05, **p<0.01, ***<0.001
