# Supplementary material for: Associations of heavy metals and urinary sodium excretion with obesity in adults: A cross-sectional study from Korean Health Examination and Nutritional Survey
Source: PLoS One. 2025 Jan 31;20(1):e0317190. doi: 10.1371/journal.pone.0317190 (PMC11785309; doi:10.1371/journal.pone.0317190)
Supplement: S4 Table — (DOCX) [file pone.0317190.s004.docx]

**Supplementary table 4.** Evaluation of Multicollinearity through Variance Inflation Factor (VIF)

| **Variables** | **Variance inflation** |
| --- | --- |
| Age | 1.083 |
| Sex | 1.151 |
| Dietary potassium intake | 1.839 |
| Dietary water intake | 1.805 |
| Dietary energy intake | 1.559 |
| Hypertension | 1.029 |
| Diabetes | 1.189 |
| Physical activity | 1.197 |
| Urinary sodium excretion | 1.044 |
| Serum mercury levels | 1.079 |
