# Supplementary material for: Associations of heavy metals and urinary sodium excretion with obesity in adults: A cross-sectional study from Korean Health Examination and Nutritional Survey
Source: PLoS One. 2025 Jan 31;20(1):e0317190. doi: 10.1371/journal.pone.0317190 (PMC11785309; doi:10.1371/journal.pone.0317190)
Supplement: S5 Table — (DOCX) [file pone.0317190.s005.docx]

**Supplementary table 5.** Linear regression Analysis of Heavy Metals and Urinary Sodium Excretion Levels with BMI (continuous variables).

| **Variables** | **β** | **Standard Error** | **P-value** |
| --- | --- | --- | --- |
| Serum cadmium levels (µg/L) | 0.361 | 0.059 | 0.08 |
| Serum mercury levels (µg/L) | 0.105 | 0.009 | <0.01 |
| Urinary arsenic excretion levels (mcg/L) | 0.001 | 0.003 | 0.62 |
| Urinary arsenic-creatinine ratio (µg /mg) | -0.013 | 0.019 | 0.47 |
| Urinary 24-hour sodium excretion levels (mg/day) | 0.009 | 0.004 | <0.01 |
