# Supplementary material for: Associations of heavy metals and urinary sodium excretion with obesity in adults: A cross-sectional study from Korean Health Examination and Nutritional Survey
Source: PLoS One. 2025 Jan 31;20(1):e0317190. doi: 10.1371/journal.pone.0317190 (PMC11785309; doi:10.1371/journal.pone.0317190)
Supplement: S6 Table — (DOCX) [file pone.0317190.s006.docx]

**Supplementary table 6.** Logistic Regression Analysis of Heavy Metal Exposure and Urinary Sodium Levels in Relation to BMI including Estimates, Standard Errors, and p-values

|  | **Underweight** | | | **Overweight & Obesity** | | |
| --- | --- | --- | --- | --- | --- | --- |
|  | Estimate | SE | P-value | Estimate | SE | P-value |
| **Serum cadmium levels (µg/L)** | | | | | | |
| Low (< 1.0) | Ref | Ref | Ref | Ref | Ref | Ref |
| High (≥ 1.0) | -0.11 | 0.09 | 0.22 | 0.03 | 0.04 | 0.45 |
| 1T (< 0.7) | Ref | Ref | Ref | Ref | Ref | Ref |
| 2T (0.7-1.3) | -0.13 | 0.12 | 0.29 | 0.03 | 0.05 | 0.54 |
| 3T (≥ 1.4) | -0.03 | 0.15 | 0.78 | 0.01 | 0.05 | 0.89 |
| **Serum mercury levels (µg/L)** | | | | | | |
| Low (< 3.9) | Ref | Ref | Ref | Ref | Ref | Ref |
| High (≥ 3.9) | -0.01 | 0.09 | 0.87 | 0.21 | 0.03 | **<0.01** |
| 1T (< 3.0) | Ref | Ref | Ref | Ref | Ref | Ref |
| 2T (3.0-5.0) | 0.14 | 0.11 | 0.22 | -0.08 | 0.05 | 0.09 |
| 3T (≥ 5.1) | -0.17 | 0.15 | 0.24 | 0.27 | 0.05 | **<0.01** |
| **Urinary arsenic excretion levels (mcg/L)** | | | | | | |
| Low (< 111.4) | Ref | Ref | Ref | Ref | Ref | Ref |
| High (≥ 111.4) | 0.09 | 0.12 | 0.45 | -0.01 | 0.05 | 0.94 |
| 1T (< 80.1) | Ref | Ref | Ref | Ref | Ref | Ref |
| 2T (80.1 – 154.3) | 0.17 | 0.17 | 0.29 | -0.01 | 0.07 | 0.86 |
| 3T (≥ 154.3) | -0.02 | 0.18 | 0.90 | -0.05 | 0.07 | 0.48 |
| **Urinary arsenic-creatinine ratio (µg /mg)** | | | | | | |
| Low (< 0.8) | Ref | Ref | Ref | Ref | Ref | Ref |
| High (≥ 0.8) | 0.02 | 0.11 | 0.88 | -0.01 | 0.05 | 0.88 |
| 1T (< 0.5) | Ref | Ref | Ref | Ref | Ref | Ref |
| 2T (0.5 – 1.3) | -0.11 | 0.16 | 0.51 | 0.04 | 0.18 | 0.59 |
| 3T (≥ 1.3) | 0.07 | 0.18 | 0.69 | -0.10 | 0.07 | 0.21 |
| **Urinary 24-hour sodium excretion levels (mg/day)** ^a^ | | | | | | |
| Low (< 3233.6) | Ref | Ref | Ref | Ref | Ref | Ref |
| High (≥ 3233.6) | -0.28 | 0.09 | **<0.01** | 0.28 | 0.04 | **<0.01** |
| 1T (< 2885.6) | Ref | Ref | Ref | Ref | Ref | Ref |
| 2T (2885.6 – 3588.7) | -0.19 | 0.13 | 0.14 | -0.07 | 0.05 | 0.17 |
| 3T (≥ 3588.8) | -0.33 | 0.15 | **0.03** | 0.43 | 0.05 | **<0.01** |

Abbreviation: T, Tertile; SE, Standard error.

a. Urinary 24-hour sodium excretion levels were estimated by Tanaka equation in a spot urine.

b. The Korean Health Examination and Nutritional Survey [KHEANS], 2008-2012
