# Supplementary material for: Associations of heavy metals and urinary sodium excretion with obesity in adults: A cross-sectional study from Korean Health Examination and Nutritional Survey
Source: PLoS One. 2025 Jan 31;20(1):e0317190. doi: 10.1371/journal.pone.0317190 (PMC11785309; doi:10.1371/journal.pone.0317190)
Supplement: Attachment — Submitted filename: Response to reviewers.pdf [file pone.0317190.s007.pdf]

We would like to thank you and the reviewers the **<u>“PLOS ONE”</u>** for taking the time to review our article **<u>(PONE-D-24-39306)</u>**. We have made some corrections and clarifications in the manuscript after going over the reviewer’s comments. The changes are summarized below: Responses to reviewer’s comments.

<u><Reviewer 1></u>

Reviewer #1: This paper offers significant value by analyzing the association between sodium intake, heavy metals, and obesity using national data from the Korean Health Examination and Nutrition Survey (KNHANES). The study draws attention to the often-overlooked factors of sodium intake and heavy metal exposure in the context of obesity.

Major comment 1: However, similar analyses have been conducted previously. For instance, the authors themselves have already reported on the association between urinary sodium levels and overweight/central obesity using the same KNHANES data in 2018 (Lee, J., Hwang, Y., Kim, KN. et al. BMC Nutr, 2018*). Additionally, other researchers have examined the association between mercury (Hg) and obesity using KNHANES data (Eom, SY., Choi, SH., Ahn, SJ. et al. Int Arch Occup Environ Health, 2014*). There is also a meta-analysis on the link between Hg and obesity (Jeon J, Park K. Korean Journal of Community Nutrition, 2023). Therefore, the introduction of this paper needs further elaboration on the rationale for analyzing sodium and heavy metals together. Why is it necessary to study the combined effects of sodium and heavy metals in relation to obesity?

Response 1: Thank you for your valuable comment. Following your suggestion, we have carefully and thoroughly revised the introduction section.

<u>The additional content is as follows:</u>

(Introduction)

In this study, the rationale for combining sodium and heavy metals in the analysis is based on their strong association with obesity and their potential contribution to its development through shared pathological pathways, such as oxidative stress and inflammation [1-3]. Sodium metabolism is primarily regulated by the kidneys [4], which also play a key role in the excretion and accumulation of heavy metals [5], suggesting a potential interaction between these two factors. Most previous studies have focused on the individual effects of sodium or heavy metal exposure, with limited research examining their combined effects. Combined exposure to sodium and heavy metals is particularly relevant in populations with high-sodium diets and environmental pollution, making it a critical public health concern.

Major comment 2: In addition, please clarify whether the findings support a synergistic effect between urinary sodium excretion and heavy metal exposure on obesity, or whether the relationship is simply additive. Can the results of this study distinguish between these two effects?

Response 2: We fully agree with the reviewer's opinion. It's very important issue. In response, we have incorporated this point into the discussion section

Our study findings indicate that the association between urinary sodium excretion and heavy metal exposure (specifically blood mercury levels) on the risk of overweight and obesity suggests the plausibility of both synergistic and additive effects. However, the results and interaction analysis provide stronger support for the presence of synergistic effects.

(1) Evidence for synergistic effects

According to the results presented in Table 3, participants with high blood mercury levels and high urinary sodium excretion exhibited a 3.61-fold higher risk of being overweight and obesity (OR = 3.61, 95% CI = 2.61–5.00) compared to those with low levels of both exposures.
The interaction analysis (p-interaction) showed statistical significance for overweight and obesity ($p < 0.01$). These findings indicate that the combined effects of the two factors exceed simple additive effects, suggesting a synergistic interaction.

(2) Potential for additive effects

Our study findings also suggest that the possibility of sodium and mercury independently increase the risk of overweight and obesity, as supported by individual analyses. Specifically, high urinary sodium excretion (≥3588 mg/day) was associated with an elevated risk of overweight and obesity (OR = 2.21, 95% CI = 1.84–2.66). Similarly, high blood mercury levels (≥ 5.2 µg/L) were associated with an increased risk of overweight and obesity (OR = 1.57, 95% CI = 1.31–1.88). These findings indicate that both exposures may independently contribute to an elevated risk of overweight and obesity.

(3) Evidence Supporting Synergistic Effects

The statistically significant p-values for interaction terms (overweight and obesity: $p < 0.01$) indicate that the combined effect of urinary sodium excretion and blood mercury levels on overweight and obesity risk exceeds their additive independent effects. Participants with high levels of both exposures had a significantly increased risk of being overweight and obesity (OR = 3.61, 95% CI = 2.61–5.00), strongly supporting the presence of a synergistic interaction.

<u>The additional content is as follows:</u>

<u>(Discussions)</u>

Our study suggests a synergistic effect between urinary sodium excretion and blood mercury levels on overweight and obesity risk, supported by statistically significant interactions. However, the cross-sectional study design limits the ability to establish causality, and quantitative assessments of the synergistic effect, such as calculating synergy indices, could not be performed. Further research is needed to confirm and elucidate these interactions.

Major comment 3: Please explain the interaction analysis (p-interaction) shown in Table 3 in the Methods section.

Response 3: We fully agree with your opinion. Per your suggestion, we revised the method section.

The additional content is as follows:

(Methods)

Additionally, we performed an interaction analysis to evaluate whether the combined effects of urinary sodium excretion and serum mercury levels on overweight and obesity exceeded the sum of their individual effects. Multiplicative interaction was assessed by calculating the p-value for the interaction term in the logistic regression model. A p-interaction value of less than 0.05 was considered statistically significant, indicating that the combination of urinary sodium and mercury has a synergistic effect on overweight and obesity risk.

Major comment 3: Additionally, the results of "We examined multi-collinearity between independent variables with Pearson correlation coefficient and Variance inflation factor." are not presented in the manuscript.

Response 3: We apologize for the errors and have added the Results section and Supplementary Tables 1 and 2.

The additional content is as follows:

(Results)

The correlation matrix (Supplementary Table 1) showed statistically significant relationships between urinary sodium excretion, blood mercury levels, and BMI ($p < 0.001$). Additionally, the multicollinearity analysis using the Variance Inflation Factor (VIF) (Supplementary Table 2) indicated that all independent variables had VIF values below 2.0, confirming no significant multicollinearity.

Minor comment 1: Study Population

Clarify whether the study population included only adults or also included children and adolescents. Table 1 mentions the exclusion of participants with chronic kidney failure and chronic liver disease, which should also be explicitly stated in the Materials and Methods section. Given the use of urinary sodium and arsenic analysis, excluding participants with chronic kidney disease is appropriate.

Response 1: Thank you for pointing out that the described contents are unclear. We revised the methods section.

<u>The revised content is as follows:</u>

(Method)

The current cross-sectional study was restricted to participants aged ≥ 19 years who completed the health examination survey, including heavy metal measurements (n = 12,000). Among these, we excluded participants with a history of stomach cancer, liver cancer, colorectal cancer, lung cancer, stroke, or cardiovascular disease, as well as those with chronic kidney failure or chronic liver diseases at enrollment (N = 1,350). These participants were excluded due to their higher tendency to develop anorexia, which could lead to unhealthy nutrient intake patterns. Additionally, participants with chronic kidney disease were excluded to ensure the accuracy of urinary sodium and arsenic analyses. Furthermore, we excluded 4,041 participants with missing information on exposure variables (spot serum and urine) and the outcome variables, including anthropometric measurements. Therefore, the final study population consisted of 6,609 participants.

Minor comment 2: Since this study primarily used calculated 24-hour urinary sodium excretion levels, please provide the formula used in the Materials and Methods section.

Response 2: We appreciate the reviewer's feedback and have incorporated it into the Methods section.

<u>The added content is as follows:</u>

(Methods)

The KNHANES provided data on urine creatinine and sodium concentrations obtained from overnight spot urine samples [6], but did not include measurements of 24-hour urinary sodium excretion levels. While 24-hour urinary sodium excretion is widely recognized as the most accurate method for assessing dietary sodium intake [7, 8], collecting 24-hour urine samples in large-scale population studies poses practical challenges. This method is prone to inaccuracies, often resulting from incomplete collection by participants. In the present study, we estimated calculated 24-h urinary sodium excretion levels using the following Tanaka's formulas (Equation 1 and 2) [9].

Equation 1) 24-h urinary creatinine excretion (mg)

$$= -2.04 \times \text{Age (year)} + 14.89 \times weight\ (kg) + 16.14 \times height\ (cm) - 2244.45.$$

Equation 2) Calculated 24-h urinary sodium excretion (mg)

$$= 21.98 \times XNA^{0.392}.$$

$\text{XNA} = 12 \times 24 - \text{h urinary creatinine excretion (mg)}$

Minor comment 3:

- If dietary potassium and dietary energy intake were adjusted for in the logistic regression models, it would be helpful to include these variables in Table 1.
- For continuous variables like sodium, arsenic (As), cadmium (Cd), and mercury (Hg), presenting the data as median (IQR) might be more appropriate.
- There is a discrepancy between the p-value for arsenic in Table 1 and the results discussed in the main text. Please correct this inconsistency.

Response 3: We appreciate the reviewer's comments and have revised Table 1 and the results accordingly.

The revised content is as follows:

(Results)

Compared with underweight and normal participants, those with overweight and obesity were more likely to be older, male, physically inactive, smokers, married, and have higher urinary sodium excretion levels, serum mercury levels, dietary energy and potassium intake, and a greater prevalence of hypertension and diabetes mellitus.

Minor comment 4: The table 2 appears overly complex. If you have performed logistic regression for underweight, overweight, and obesity groups, the "normal" column may not be necessary. Instead, consider focusing on logistic regression analysis for "obesity" or a combined "overweight+obesity" group.

Response 4: Thank you for your insightful comments. Following your suggestion, we reanalyzed all tables by combining the "overweight+obesity" group. This approach better aligns with the study's objectives and simplifies the interpretation. Additionally, we have revised the overall results of the manuscript.

Minor comment 5: Additionally, the "Total" column seems redundant and could be removed.

Response 5: We fully agree with your opinion. As per your suggestion, the "Total" column has been removed from Table 2.

Minor comment 6: If you categorized the variables (low/high) based on the median values, please describe this in the Methods section or Table footnote.

Response 6: We agree with the reviewer's point and have clarified the process of categorizing variables (e.g., low/high based on median values and tertiles) by providing additional descriptions in the Methods section.

<u>The added content is as follows:</u>

(Methods)

This study categorized continuous variables, including serum cadmium, serum mercury, urinary arsenic, urinary arsenic-creatinine ratio, and urinary 24-hour sodium excretion levels, into low and high groups based on their median values. Furthermore, each variable was further divided into tertiles (1T, 2T, and 3T) to conduct additional analyses.

Minor comment 6: In Table 1, variables such as smoking status, marital status, income, and education showed significant differences across BMI categories. It is unclear why these variables were not adjusted for in the regression models. Please clarify this choice.

Response 6: We fully agree with the reviewer's opinion. Following your suggestion, we have incorporated additional controls for confounding factors, including smoking status, income, marital status, and education level, into the previous logistic model, as reflected in Table 2, Table 3, and Supplementary Tables 1 and 2.

Minor comment 7: Figure 1 is not referenced in the main text, and there is a mismatch between the numbers in Figure 1 and those provided in the Methods section. Please ensure consistency between the figures and the text.

Response 7: We apologize for the errors, and we have corrected the methods section and figure 1.

<u>The revised sentences are as follows:</u>

(Methods)

The current cross-sectional study was restricted to participants aged ≥ 19 years who completed the health examination survey, including heavy metal measurements (n = 12,000). Among these, we excluded

participants with a history of stomach cancer, liver cancer, colorectal cancer, lung cancer, stroke, or cardiovascular disease, as well as those with chronic kidney failure or chronic liver diseases at enrollment (N = 1,350). These participants were excluded due to their higher tendency to develop anorexia, which could lead to unhealthy nutrient intake patterns. Additionally, participants with chronic kidney disease were excluded to ensure the accuracy of urinary sodium and arsenic analyses. Furthermore, we excluded 4,041 participants with missing information on exposure variables (spot serum and urine) and the outcome variables, including anthropometric measurements.

<Reviewer 2>

Reviewer #2: This is a really interesting and important manuscript. The authors tried there best to describe the entire process so that reader can get the take home message and can replicate similar studies. But to consider it for publishing, a major revision is required. The following are the overall areas where the authors should focus and submit the revised version.

Major comment 1: The research objective was not clearly stated. Is this study to identify novel risk factors? or just to explore the association between heavy metal, urinary sodium excretion and obesity and overweight. The author should clearly describe it.

Response 1: Thank you for pointing out that the described contents are unclear. We revised the introduction section.

The revised sentences are as follows:

(Introduction)

Therefore, this study aimed to investigate the relationship between urinary sodium excretion and heavy metal exposures (cadmium, mercury, and arsenic) in overweight and obesity, focusing on their individual effects. Additionally, this study aims to evaluate the combined effects of urinary sodium excretion and serum mercury levels on the risk of overweight and obesity, exploring potential interactive effects between these exposure factors.

Major comment 2: The author mentioned that the aim was to identify novel risk factors (line 82-83)... but in the result the author didn't highlight what was/were the novel risk factor and why that is novel?

Response 2: Thank you for pointing out that the described contents are unclear. To clarify the aim of the study, we have removed the term 'novel risk'.

Major comment 3: In the statistical analysis section, the author mentioned that, they used polytomous logistic regression model but in the result section reported results on spline. These needs to be clearly explained.

Response 3: We appreciate the reviewer's comments and have revised the methods section to explain this more clearly.

The revised sentences are as follows:

(Methods)

We utilized a polytomous logistic regression model to analyze the relationship between heavy metals, urinary sodium excretion, and overweight and obesity. Regression analyses incorporating continuous variables were also conducted. Additionally, restricted cubic splines were employed to visualize potential non-linear associations.

Major comment 4: Moreover, there is a lack of description of the outcome (dependent) variable for the logistic regression model. The statistical analysis part should be revisited with clearly defined the dependent and covariates for the model and perform the analysis accordingly.

Response 4: We sincerely appreciate the reviewer's valuable comments. We have revised the statistical analysis section to provide a detailed explanation of the dependent variable used in the model. Furthermore, we have added a covariate section to elaborate on the details.

The added sentences are as follows:

(Methods)

For this study, the dependent variable was defined as a categorical variable with three levels: underweight, normal weight, overweight, and obesity, based on BMI thresholds (underweight: BMI < 18.5 kg/m²; normal: 18.5 ≤ BMI < 23 kg/m²; overweight and obesity: BMI ≥ 23 kg/m²).

Covariate

Information on sociodemographic factors such as age, sex, household income, education level, marital status, dietary energy, and potassium intake, as well as health-related factors including smoking habits, physical activity, hypertension, and diabetes mellitus, was gathered through medical examinations and self-reported questionnaires. Household income levels were divided into quartiles, ranging from quartile 1 (low) to quartile 4 (high). Education status was categorized as below middle school, high school, college, and higher than university. Marital status was defined as married or single. Drinking habit was classified as yes or no. Participants were also categorized by their smoking status as non-smokers or smokers. Moderate physical activity was recorded as yes or no. Hypertension was defined as the presence of high blood pressure or the use of antihypertensive medication, categorized as yes or no. Diabetes mellitus was categorized as yes or no, based on a history of the condition or diagnostic criteria (e.g., fasting glucose ≥ 126 mg/dL). Dietary energy intake was recorded in kilocalories per day (kcal/day), and dietary potassium intake was recorded in milligrams per day (mg/day).

Major comment 5: Also, it is highly recommended to address clustering effect due to sampling techniques. A robust standard error should be computed and all the inference should be upon robust standard errors.

Response 5: We sincerely appreciate the reviewer's insightful comments. This study accounted for the complex sampling design of the Korea National Health and Nutrition Examination Survey (KNHANES) by incorporating stratification variables, cluster variables, and sampling weights into the analysis using PROC SURVEYLOGISTIC. Robust standard errors were calculated to address the clustering effect inherent in the sampling design. These details have been added to the Statistical Analysis section. All statistical inferences were based on robust standard errors, and the results are presented in Supplementary Table 6.

The added sentences are as follows:

(Methods)

This study adhered to the KNHANES data analysis guidelines provided by the KCDC. KNHANES utilizes a complex sampling design incorporating stratification, cluster sampling, and sampling weights. Accordingly, PROC SURVEYLOGISTIC was employed to conduct analyses accounting for this complex sampling design.

Major comment 4: I would also recommend to do a linear regression without categorising the body weight variable and add that as a supplementary information.

Response 4: We fully agree with the reviewer's opinion. We performed further analysis (Supplementary table 5).

The added sentences are as follows:

(Results)

These associations were consistent with the results of the linear regression analysis (Supplementary table 5).

Major comment 5: The tables should be revisited, the message from the table is not easy to understand by readers. It should be revisited.

Response 5: We agree with the reviewer's point. Following your suggestion, we have revised the tables.

Minor comment 1: As a minor comments, the author should add more recent work related to urinary sodium excretion and obesity. There are recent publication out there that are more recent that the author cited in this manuscript.

Response 1: Thank you for your valuable comment. As suggested, we have added references to recent studies related to urinary sodium excretion and obesity.

Minor comment 2: Line 65: Recent investigations have suggested a potential link between heavy metal exposure and obesity. This statement should supported by results and a proper citation.

Response 2: We apologize for the errors, and we have included appropriate citations to support the statement.

The revised sentences are as follows:

(Introduction)

Emerging evidence also suggests a potential connection between heavy metal exposure and obesity [10, 11].

Table 1. General characteristics of study subject, Korea National Health Examination and Nutritional Survey (KNHANES), 2008-2012.

	**Normal (N=4,225)**	**Underweight (N=291)**	**Overweight & Obesity (N=2,093)**	**P-value**
	N (%)	N (%)	N (%)	
Sex				
Male	2053 (61.2)	93 (2.8)	1206 (36.0)	<0.01
Female	2172 (66.7)	198 (6.1)	887 (27.2)	
Monthly household income (KRW)				
1Q (< 1,500,000)	1031 (63.2)	67 (4.1)	533 (32.7)	0.04
2Q (1,500,000–2,999,000)	1023 (61.9)	64 (3.9)	565 (34.2)	
3Q (3,000,000–3,999,000)	1056 (65.5)	83 (5.1)	473 (29.3)	
4Q (≥ 4,000,000)	1071 (65.2)	75 (4.6)	496 (30.2)	
Education status				
Below Middle school	1814 (61.7)	71 (2.4)	1053 (35.8)	<0.01
High school	973 (68.5)	65 (4.6)	383 (26.9)	
College	840 (64.0)	85 (6.5)	387 (29.5)	
Higher than University	581 (63.4)	69 (7.5)	266 (29.0)	
Marital status				
Married	3282 (62.8)	164 (3.1)	1780 (34.1)	<0.01
Single	936 (68.2)	127 (9.2)	310 (22.6)	
Drinking habit				
No	458 (62.6)	28 (3.8)	246 (33.6)	0.42
Yes	3742 (64.1)	258 (4.4)	1837 (31.5)	
Smoking habits				
No	2294 (65.7)	188 (5.4)	1011 (28.9)	<0.01
Yes	1900 (61.9)	98 (3.2)	1072 (34.9)	
Moderate physical activity				
No	2712 (65.0)	209 (5.0)	1251 (30.0)	<0.01
Yes	1490 (62.1)	78 (3.3)	832 (34.7)	
Hypertension				
No	3279 (68.6)	270 (5.6)	1231 (25.7)	<0.01
Yes	944 (51.7)	21 (1.2)	860 (47.1)	
Diabetes mellitus				
No	3244 (68.2)	256 (5.4)	1259 (26.5)	<0.01
Yes	855 (51.6)	24 (1.5)	779 (47.0)	
	**Median (IQR)**	**Median (IQR)**	**Median (IQR)**	
Age (years)	44 (24)	31 (16)	47 (22)	<0.01
Urinary sodium excretion levels (mg/day) [a]	3170.4 (1044.3)	2692.9 (978.8)	3445.6 (1148.9)	<0.01
Urinary arsenic excretion levels (mcg/L)	111.7 (122.5)	101.6 (91.4)	113.2 (120.1)	0.14
Urinary arsenic-creatinine ratio (µg /mg)	0.8 (1.5)	0.6 (1.3)	0.8 (1.4)	0.25
Serum cadmium levels (µg/L)	1.0 (0.7)	0.8 (0.7)	1.1 (0.7)	<0.01
Serum mercury levels (µg/L)	3.8 (2.9)	3.1 (2.0)	4.5 (3.7)	<0.01
Dietary energy intake (mg/day)	2005.5 (1442.8)	1828.3 (1263.8)	2085.2 (1508.1)	<0.01
Dietary potassium intake (mg/day)	3107.1 (2556.5)	2602.4 (2154.0)	3259.3 (2762.9)	<0.01

a. Urinary 24-hour sodium excretion levels were estimated by Tanaka equation in a spot urine.

Table 2. Odds Ratios (OR) and 95% Confidence Intervals (CI) for Overweight and Obesity Based on Levels of Heavy Metals and 24-Hour Sodium Excretion in the Healthy General Population (N = 6,609)[a]

	**Normal (N=4,225)**	**Underweight (N=291)**	**Underweight**	**Overweight & Obesity (N=2,093)**	**Overweight & Obesity**
	N (%)	N (%)	OR (95% CI)[b]	N (%)	OR (95% CI)[b]
**Serum cadmium levels (µg/L)**					
Low (< 1.0)	2127 (50.3)	190 (65.3)	Ref	986 (47.1)	Ref
High (≥ 1.0)	2098 (49.7)	101 (34.7)	0.80 (0.56-1.14)	1107 (52.9)	1.06 (0.91-1.24)
1T (< 0.7)	1415 (33.5)	143 (49.1)	Ref	640 (30.6)	Ref
2T (0.7-1.3)	1241 (33.6)	80 (27.5)	0.75 (0.52-1.08)	709 (33.9)	1.07 (0.90-1.28)
3T (≥ 1.4)	1389 (32.9)	68 (23.4)	0.82 (0.61-1.30)	744 (35.5)	1.05 (0.87-1.25)
**Serum mercury levels (µg/L)**					
Low (< 3.9)	2252 (53.3)	199 (68.4)	Ref	852 (40.7)	Ref
High (≥ 3.9)	1973 (46.7)	92 (31.6)	0.97 (0.68-1.39)	1241 (59.3)	**1.51 (1.31-1.75)**
1T (< 3.0)	1492 (35.3)	141 (48.5)	Ref	564 (26.9)	Ref
2T (3.0-5.0)	1448 (34.3)	103 (35.4)	1.10 (0.80-1.52)	660 (31.5)	1.10 (0.92-1.32)
3T (≥ 5.1)	1285 (30.4)	47 (16.2)	0.81 (0.50-1.29)	869 (41.5)	**1.57 (1.31-1.88)**
**Urinary arsenic excretion levels (mcg/L)**					
Low (< 111.4)	962 (49.9)	83 (56.5)	Ref	448 (49.1)	Ref
High (≥ 111.4)	966 (50.1)	64 (43.5)	1.20 (0.74-1.94)	464 (50.9)	0.99 (0.82-1.20)
1T (< 80.1)	632 (32.8)	54 (36.7)	Ref	308 (33.8)	Ref
2T (80.1 – 154.3)	636 (33.0)	56 (38.1)	1.38 (0.81-2.36)	306 (33.6)	0.92 (0.72-1.19)
3T (≥ 154.3)	660 (34.2)	37 (25.2)	1.14 (0.61-2.10)	298 (32.7)	0.89 (0.69-1.14)
**Urinary arsenic-creatinine ratio (µg /mg)**					
Low (< 0.8)	959 (49.7)	82 (55.8)	Ref	453 (49.7)	Ref
High (≥ 0.8)	969 (50.3)	65 (44.2)	1.04 (0.65-1.65)	459 (50.3)	1.00 (0.79-1.22)
1T (< 0.5)	621 (32.2)	66 (44.9)	Ref	307 (33.7)	Ref
2T (0.5 – 1.3)	649 (33.7)	37 (25.2)	0.86 (0.51-1.46)	312 (34.2)	0.98 (0.77-1.26)
3T (≥ 1.3)	658 (34.1)	44 (29.9)	1.03 (0.58-1.83)	293 (32.1)	0.86 (0.67-1.11)
**Urinary 24-hour sodium excretion levels (mg/day)** [c]					
Low (< 3233.6)	2247 (53.2)	281 (74.9)	Ref	839 (40.1)	Ref
High (≥ 3233.6)	1978 (46.8)	73 (25.1)	**0.57 (0.40-0.82)**	1254 (59.9)	**1.76 (1.51-2.05)**
1T (< 2885.6)	1505 (35.6)	182 (62.5)	Ref	513 (24.5)	Ref
2T (2885.6 – 3588.7)	1453 (34.4)	70 (24.1)	**0.49 (0.34-0.70)**	685 (32.7)	**1.34 (1.11-1.61)**
3T (≥ 3588.8)	1267 (30.0)	39 (13.4)	**0.42 (0.27-0.67)**	895 (42.8)	**2.21 (1.84-2.66)**

Abbreviation: T, Tertile; OR, Odds ratio.

a. Analyses are adjusted for stratification, clustering, and sampling weights. Normal weight serves as the reference category.

b. Adjusted for age, sex, household income, education level, marital status, smoking habits, moderate physical activity, dietary potassium, dietary energy intake, history of diabetes and hypertension.

c. Urinary 24-hour sodium excretion levels were estimated by Tanaka equation in a spot urine.

Table 3. OR (95% CI) of overweight and obesity by the combination of serum mercury and urinary 24-hour sodium excretion in the general population (N = 6,609) [a]

**Urinary sodium levels** [c]	**Serum mercury levels**	**Normal (N=4,225)**	**Underweight (N=291)**	**Underweight**	**Overweight & Obesity (N=2,093)**	**Overweight & Obesity**
		N (%)	N (%)	OR (95%CI) [b]	N (%)	OR(95%CI) [b]
1T	1T	591 (14.0)	93 (32.0)	Ref	143 (6.8)	Ref
2T	1T	496 (11.7)	59 (20.3)	1.01 (0.64-1.61)	164 (7.8)	1.22 (0.85-1.75)
1T	2T	503 (11.9)	32 (11.0)	0.92 (0.51-1.68)	206 (9.8)	**1.64 (1.16-2.32)**
2T	2T	490 (11.6)	26 (8.9)	**0.49 (0.29-0.81)**	202 (9.6)	**1.56 (1.10-2.21)**
3T	1T	418 (9.9)	30 (10.3)	**0.54 (0.29-0.99)**	206 (9.8)	*1.40 (0.98-2.01)*
1T	3T	399 (9.4)	16 (5.5)	**0.38 (0.17-0.84)**	277 (13.2)	**2.17 (1.53-3.08)**
3T	2T	461 (10.9)	12 (4.1)	**0.32 (0.16-0.68)**	219 (10.5)	**2.27 (1.59-3.23)**
2T	3T	462 (10.9)	18 (6.2)	0.64 (0.45-1.21)	290 (13.9)	**2.58 (1.86-3.59)**
3T	3T	406 (9.6)	5 (1.7)	**0.23 (0.06-0.88)**	386 (18.4)	**3.61 (2.61-5.00)** [d]
Low	Low	1251 (29.6)	151 (51.9)	Ref	342 (16.3)	Ref
	High	996 (23.6)	67 (23.0)	1.07 (0.69-1.65)	497 (23.7)	**1.67 (1.35-2.08)**
High	Low	1001 (23.7)	48 (16.5)	**0.63 (0.41-0.97)**	510 (24.4)	**1.93 (1.54-2.43)**
	High	977 (23.1)	25 (8.6)	**0.51 (0.28-0.93)**	744 (35.6)	**2.69 (2.17-3.34)**

Abbreviation: T, Tertile ; OR, Odds ratio.

a. Analyses are adjusted for stratification, clustering, and sampling weights. Normal weight serves as the reference category.

b. Adjusted for age, sex, household income, education level, marital status, smoking habits, moderate physical activity, dietary potassium, dietary energy intake, history of diabetes and hypertension.

c. Urinary 24-hour sodium excretion levels were estimated by Tanaka equation in a spot urine.

d. P-interaction: $p < 0.01$

<Reference>

1. Vincent HK, Innes KE, Vincent KR. Oxidative stress and potential interventions to reduce oxidative stress in overweight and obesity. Diabetes, obesity and metabolism. 2007;9(6):813-39.
2. Ertuglu LA, Sahinoz M, Alsouqi A, Deger SM, Guide A, Stewart TG, et al. High tissue-sodium associates with systemic inflammation and insulin resistance in obese individuals. Nutrition, Metabolism and Cardiovascular Diseases. 2023;33(7):1398-406.
3. Tinkov AA, Aschner M, Ke T, Ferrer B, Zhou J-C, Chang J-S, et al. Adipotropic effects of heavy metals and their potential role in obesity. Faculty Reviews. 2021;10:32.
4. Bie P. Mechanisms of sodium balance: total body sodium, surrogate variables, and renal sodium excretion. American Journal of Physiology-Regulatory, Integrative and Comparative Physiology. 2018;315(5):R945-R62.
5. Lentini P, Zanoli L, Granata A, Signorelli SS, Castellino P, Dellaquila R. Kidney and heavy metals-The role of environmental exposure. Molecular medicine reports. 2017;15(5):3413-9.
6. Kweon S, Kim Y, Jang M-j, Kim Y, Kim K, Choi S, et al. Data resource profile: the Korea national health and nutrition examination survey (KNHANES). International journal of epidemiology. 2014;43(1):69-77.
7. Xu J, Wang M, Chen Y, Zhen B, Li J, Luan W, et al. Estimation of salt intake by 24-hour urinary sodium excretion: a cross-sectional study in Yantai, China. BMC public health. 2014;14:1-6.
8. McLean RM, Farmer VL, Nettleton A, Cameron CM, Cook NR, Campbell NR, et al. Assessment of dietary sodium intake using a food frequency questionnaire and 24-hour urinary sodium excretion: a systematic literature review. The Journal of Clinical Hypertension. 2017;19(12):1214-30.
9. Tanaka T, Okamura T, Miura K, Kadowaki T, Ueshima H, Nakagawa H, et al. A simple method to estimate populational 24-h urinary sodium and potassium excretion using a casual urine specimen. Journal of human hypertension. 2002;16(2):97-103.
10. Zangiabadian M, Jolfayi AG, Nejadghaderi SA, Amirkhosravi L, Sanjari M. The association between heavy metal exposure and obesity: A systematic review and meta-analysis. Journal of Diabetes & Metabolic Disorders. 2024;23(1):11-26.
11. Duc HN, Oh H, Kim M-S. The effect of mixture of heavy metals on obesity in individuals≥ 50 years of age. Biological trace element research. 2022;200(8):3554-71.
