# Supplementary material for: Associations of heavy metals and urinary sodium excretion with obesity in adults: A cross-sectional study from Korean Health Examination and Nutritional Survey
Source: PLoS One. 2025 Jan 31;20(1):e0317190. doi: 10.1371/journal.pone.0317190 (PMC11785309; doi:10.1371/journal.pone.0317190)
Supplement: Attachment — Submitted filename: Response to Reviewers.pdf [file pone.0317190.s008.pdf]

We would like to thank you and the reviewers the **<u>"PLOS ONE"</u>** for taking the time to review our article **<u>(PONE-D-24-39306R1)</u>**. We have made some corrections and clarifications in the manuscript after going over the reviewer's comments. The changes are summarized below: Responses to reviewer's comments.

<u><Reviewer 1></u>

The authors have adequately addressed the comments raised in the previous round of review. I believe the manuscript has improved significantly. There is still some uncertainty about whether sodium and mercury really have a synergistic effect. In Table 3, the odds ratio (OR) for the combination of sodium 3T/mercury 3T is 3.61, which is the highest. However, the OR for Sodium 3T/Mercury 1T is 1.40 and for Sodium 1T/Mercury 3T it is 2.17. The OR of 3.61 is not significantly greater than the sum of the ORs of 1.40 and 2.17. Although the reported p for interaction suggests an interaction between the two variables, this does not necessarily confirm a synergistic effect. However, the authors have appropriately acknowledged in the DISCUSSION section that further research is needed to confirm whether this represents a synergistic effect. This limitation has been well addressed and is acceptable.

Response: We appreciate the reviewer's insightful comments regarding the potential synergistic effect between urinary sodium excretion and blood mercury levels. In response, we have clarified in the manuscript that while our findings suggest a potential interaction between these variables, we were unable to conclusively demonstrate a synergistic effect. We have also noted the limitations of our study in this regard, particularly the inability to perform quantitative evaluations, such as calculating the synergy index. We have appropriately addressed this limitation in the Discussion section and emphasized the need for further research to confirm these findings. Thank you for acknowledging that this limitation has been adequately addressed.

<u>The revised content is as follows:</u>

(Methods)

A p-interaction value of less than 0.05 was considered statistically significant, indicating a potential interaction between urinary sodium excretion and mercury levels in relation to the risk of overweight and obesity.

(Discussions)

Third, our study identified a potential interaction between urinary sodium excretion and blood mercury levels in relation to the risk of overweight and obesity. However, we were unable to clearly demonstrate a synergistic effect, and further quantitative analyses, such as the calculation of a synergy index, are required to confirm these findings [1].

Minor comment 1: Line 228, In Figure 23, the relationship between urinary sodium excretion levels and overweight/obesity appears linear. Is it non-linear?

Response 1: Thank you for pointing out the errors. We have revised the Results section.

<u>The revised content is as follows:</u>

(Results)

Figure 2 illustrates the associations between heavy metals, urinary sodium excretion levels, and the odds of overweight and obesity. Serum mercury levels exhibit a potential non-linear trend, while urinary sodium excretion demonstrates a significant linear association, with higher levels correlating with increased odds of overweight and obesity.

Minor comment 2: There is an error in reporting the OR and 95% CI. The correct value should be OR 3.61 (2.61–5.00) instead of OR 0.23 (0.06–0.88). Please revise this accordingly.

Response 2: We apologize for the errors and have revised the Results section.

<u>The revised content is as follows:</u>

(Results)

Participants with high urinary sodium and high serum mercury levels (3T/3T) had significantly higher odds of overweight and obesity compared to the reference group with low urinary sodium and low serum mercury levels (1T/1T) (OR = 3.61, 95% CI = 2.61–5.00, p-interaction < 0.01).

Reference

1. Knol MJ, VanderWeele TJ, Groenwold RH, Klungel OH, Rovers MM, Grobbee DE. Estimating measures of interaction on an additive scale for preventive exposures. European journal of epidemiology. 2011;26:433-8.
